# County-level Racial/Ethnic Residential Segregation and Physical Activity Behavior among US Adults

**DOI:** 10.1007/s11524-024-00913-4

**Published:** 2024-09-10

**Authors:** Yangyang Deng, Mohammad Moniruzzaman, Breanna Rogers, Kelly K. Jones, Pedro F. Saint-Maurice, Shreya Patel, David Berrigan, Charles E. Matthews, Kosuke Tamura

**Affiliations:** 1 Socio-Spatial Determinants of Health (SSDH) Laboratory, Population and Community Health Sciences Branch, Division of Intramural Research, National Institute on Minority Health and Health Disparities, National Institutes of Health, Bethesda, MD USA; 2 Neighborhoods and Health Laboratory, Population and Community Health Sciences Branch, Division of Intramural Research, National Institute on Minority Health and Health Disparities, National Institutes of Health, Bethesda, MD USA; 3 Champalimaud Foundation, Lisbon, Portugal; 4 Metabolic Epidemiology Branch, Division of Cancer Epidemiology and Genetics, National Cancer Institute, National Institutes of Health, Rockville, MD USA; 5 Epidemiology and Biostatistics Department, Dornsife School of Public Health Drexel University, Philadelphia, PA USA; 6 Behavioral Research Program, Division of Cancer Control and Population Sciences, National Cancer Institute, National Institutes of Health, Rockville, MD USA

**Keywords:** Residential segregation, Physical activity, Domain-specific activities, Adults

## Abstract

The relationship between racial/ethnic residential segregation and physical activity (PA) remains unclear with both positive and negative associations between segregation and PA recently reported. We aimed to examine the relationship between county-level residential segregation and total daily PA and domain-specific PA and whether these associations varied by gender. Participants (*N* = 2625, mean age [SD] = 45.2 [15.4]) were recruited from the AmeriSpeak panel who completed up to two Activities Completed over Time in 24 Hours (ACT24) previous day recalls in 2019. PA outcomes were created for the following: (1) light PA (LPA), (2) moderate-to-vigorous PA (MVPA), (3) total active time, and (4) domain-specific PA, including leisure, work, household, transport, personal, and other activities. County-level residential segregation based on isolation. Weighted generalized linear models were used to examine the relationship between county-level segregation and each PA outcome, controlling for age, gender, race/ethnicity, income, employment, body mass index, county-level poverty, and census region. Overall, results showed no association between county-level residential segregation and LPA, MVPA, total active time, and domain-specific PA among NH Black and Hispanic populations. Among NH Black females, greater residential segregation was associated with more total hours/day of activity (*β* = 3.54, 95% CI [0.23, 6.85]). Only NH Black females living in segregated neighborhoods had more total active time. Additionally, it is important to acknowledge that these relationships may vary among NH Black and Hispanic populations. Future studies should examine the interaction between segregation and a broader range of individual, contextual, and environmental factors in relation to PA and domain-specific PA.

**Supplementary Information:**

The online version contains supplementary material available at 10.1007/s11524-024-00913-4.

## Introduction

Physical activity (PA) is widely recognized as a modifiable health behavior associated with the risk of developing chronic diseases [1], including cardiovascular disease (CVD), cancers, and diabetes. Higher PA levels are also related to psychological well-being, including a reduced risk for depression, anxiety, and dementia [2, 3]. Increasing levels of adherence to PA guidelines could significantly improve public health. Previous research has examined individual (i.e., gender, age, education, income) and social factors as correlates of PA among adults [4, 5], with further substantial research focused on neighborhood social and physical environments as potential determinants of PA [6–8]. However, the relationship between neighborhood structural factors, such as racial and ethnic residential segregation and PA, has received less attention.

Racial and ethnic residential segregation refers to the degree to which individuals from different racial and ethnic backgrounds live in separate neighborhoods. Residential segregation itself does not contribute to an unequal distribution of resources; rather, it is the indicator of specific policies, investments, or disinvestments initiated by local, state, and federal agencies, as well as elected officials, that contribute to both the unequal distribution of resources and the perpetuation of segregation [9]. However, repercussions of segregation lead to a variety of societal problems, such as restricted access to quality education and employment opportunities, thereby exacerbating health disparities [10–12]. For example, previous research showed a negative association between residential segregation and chronic diseases, including CVD, cancer, obesity, and diabetes [13, 14]. PA has also been demonstrated to play a protective role in such chronic diseases. However, only a few previous studies have investigated the relationship between racial and ethnic residential segregation and PA, with inconsistent findings across racial and ethnic groups [15–18]. Further exploration of this topic could help clarify these inconsistencies.

Several significant gaps remain in understanding the relationships between residential segregation and PA. First, prior research has predominantly focused on Black vs White neighborhood residential segregation, but less is known about other minority groups such as Hispanic populations [11]. Investigating beyond Black vs White residential segregation may contribute to a better understanding of various racial/ethnic minorities’ PA behavior. Second, research has consistently shown significant gender differences in PA participation by certain neighborhood characteristics (e.g., access to recreational facilities and neighborhood walkability) [19]. Furthermore, past studies have focused on overall levels of PA without examining domain-specific PA behaviors, including leisure, occupational, and transportation-related activities. Thus, we address three gaps using a county-level measure of residential segregation and domain-specific estimates of PA. Our first aim was to investigate the relationship between county-level residential segregation and various PA outcomes, including light-intensity PA (LPA), moderate-to-vigorous PA (MVPA), and total active time, stratified by racial and ethnic groups. Second, we examined the gender-specific associations between county-level residential segregation and PA outcomes. Lastly, we estimated the associations between county-level residential segregation and domain-specific PA (i.e., leisure, work, household, transport, personal, and other activities).

## Methods

### Study Population and Design

This study was based on a cross-sectional study conducted within the AmeriSpeak panel (https://www.amerispeak.org), a probability-based survey designed to represent the US population. The AmeriSpeak panel utilized a stratified two-stage sampling design (details in supplement) [20]. Data collection was completed between October and November 2019 by the National Opinion Research Center (NORC) at the University of Chicago [21]. ACT24 recalls were administered online using a link sent via email or text message. The present study included participants aged 20 to 75 who completed a short online survey and a previous-day recall on a randomly selected day. Participants who completed the first survey were invited to a second recall on a different randomly selected day, 1–2 weeks later. Our analytic sample included participants who completed the survey and provided at least one valid ACT24 recall. The NORC research ethics review board approved the study protocol, and all participants provided written informed consent. Participants received up to $30 for completing study activities.

From the total 2877 participants contributing a total of 4575 recalls, we excluded recalls based on any one of the following criteria: (1) participants reported more than 1 h per day of unknown time (gaps; private unreported time; *n* = 257 recalls), (2) more than 2 h per day of overlapping time (*n* = 93 recalls), and (3) recalls 0 active hours of PA (*n* = 87 recalls) [22]. After exclusions, the survey sample included 2625 participants with at least one valid recall (*n* = 2226 first recalls, *n* = 1912 second recalls).

### Sample Weights

The AmeriSpeak panel utilized a stratified two-stage sampling design [20]. Briefly, the first stage involved selecting National Frame Areas as primary sampling units and census tracts or block groups as secondary sampling units [20]. To approximate a nationally representative sample of the US population, sample weights were developed for each recall based on the final weights of AmeriSpeak panelists, which adjusted for panel selection probabilities, non-response, and population coverage. The final weights were adjusted to external population totals based on age, gender, race/ethnicity, housing tenure, telephone status, and Census Division [22]. The study-specific sampling weights were calculated for each recall day, aiming to address potential variations in PA levels throughout the week. Lastly, the weights were normalized to ensure that each day of the week contributed equally to the analyses. This sampling strategy allowed us to obtain a representative sample of US adults and estimate PA levels in the population across different days of the week [20].

### PA Outcomes

Data were collected using the self-administered ACT24 previous-day recall, which asked participants to report their PA via smartphone, tablet, or computer for the previous day (midnight-midnight). Recalls with less than one hour of missing or unknown activity (e.g., gap time, private, prefer not to say time) and at least 22 h of total reported time were considered valid. ACT24 has shown greater accuracy and higher internal correlations than a traditional questionnaire in large epidemiologic studies. Participants could select from 170 individual activities grouped into 14 major categories linked to the Compendium of Physical Activities [23, 24]. Each activity was assigned a score to estimate energy expenditure in metabolic equivalent of tasks (METs) mins/day. Reportable PA behaviors included light (1.8–2.9 METs), moderate (3.0–5.9 METs), and vigorous intensity (≥ 6.0 METs) activities [25]. Subsequently, three PA outcomes (hours/day [h/d]) were created: LPA, MVPA, and total active time (including LPA and MVPA). Consistent with the American Time-use Survey [26], time-use PA outcomes were calculated for seven domains-specific PA (h/d), including leisure, work, household, transportation, personal, and other activities.

### County-level Racial Residential Indices

We utilized a widely accepted segregation index to measure the degree to which different racial and ethnic groups are isolated from one another within their county. The calculated isolation index values were merged with the respective participant’s Federal Information Processing Standards (FIPS) codes at the county level.

### Isolation Index

$$\text{Isolation}= {\sum }_{i=1}^{n}\left[\frac{{x}_{i}}{X}\right]\times \left[\frac{{x}_{i}}{{t}_{i}}\right]$$

where *x*_*i*_ is the number of residents who are NH Black/Hispanic individuals, the number of residents who are NH Black/Hispanic in census tract *i*, t_*i*_ is the total population in tract *i*. *X* is the number of NH Blacks/Hispanic people in the county. The isolation index measures the degree to which members of a minority group are primarily exposed to other members of the same group, based on residence in a specific area, as opposed to members of other racial or ethnic groups [27, 28]. In this study, we calculated the index based on the county-level of residence. A higher index value indicates a greater probability of contact between members of the same minority group (high segregation) and a lower probability of contact between members of that group and individuals from other racial or ethnic groups. This analysis includes the following racial/ethnic isolation segregation indices: NH Black versus others (including all other race/ethnicity groups such as NH White, Hispanic, Asian, and others), and Hispanic versus others (including all other race/ethnicity groups such as NH White, NH Black, Asian, and other racial and ethnic groups).

### Covariates

Our analysis incorporated individual-level covariates that could influence the associations between the segregation indices and the PA outcomes. Demographic covariates included age, gender (male/female), educational attainment (high school or less, some college/associate degree, bachelor’s degree, or graduate degree), and household income: “ < 50,000,” “50,000–99,000,” “100,000–149,000,” and “150,000 + ” [22]. BMI was coded as a continuous variable.

Additional neighborhood-level variables could be associated with the exposure and outcome variables. County-level poverty was indicated as the percentage of households living below the poverty line within each county (expressed in quartiles), with a higher quartile indicating higher poverty levels. Region included four census regions: Northeast, Midwest, South, and West.

### Statistical Analysis

Participants’ characteristics were summarized for the overall sample and stratified by gender. Each isolation segregation index was modeled as a continuous variable. For continuous variables, we calculated weighted means and standard deviations (SD). To describe PA behavior, we used data from one recall, up to two recalls per person, focusing on PA behavior for days rather than individuals. We reported the mean PA outcomes and domain-specific PA for the overall population and stratified by gender.

We conducted weighted multivariate linear regression to examine the association between isolation segregation indices and PA outcomes (h/d, including LPA, MVPA, and total active time) stratified by racial and ethnic groups. We set two-tailed *p*-values < 0.05 as the threshold for statistical significance across all analyses. Furthermore, we investigated whether the association is moderated by gender for NH Black and Hispanic groups separately. Lastly, we separately examined the associations between segregation and each domain-specific PA for NH Black and Hispanic groups. All analyses were adjusted for covariates. Additionally, we calculate NH Black and Hispanic isolation indices using NH White adults as the reference group for the sensitivity analysis. To account for the complex sampling design, we performed analyses using the “Survey” package in the R statistical software (version 4.2.2; www.r-project.org).

## Results

### Descriptive Statistics

A total of 2625 participants (mean age [SD] = 45.2 [15.4]) with 4138 recalls from the AmeriSpeak panel completed up to two ACT24 recalls in 2019 (Table 1). 50.6% of the participants were female, and 49.4% were males. 63.7% of the participants identified as NH White adults, followed by 16.8% Hispanic, 10.9% NH Black, and 8.6% other adults. Among participants, 34.2% had a high school diploma or less, 40.8% earned less than $50,000, 67.3% were employed, and 38.2% were from the South. Quartile county-level poverty ranged from 29.7% in quartile one (highest poverty) to 7.1% in quartile four (wealthiest).

**Table 1:** Weighted descriptive characteristics of the study participants

Characteristics	All	Male	Female
	*n* = 2625	*n* = 1448 (49.4%)	*n* = 1177 (50.6%)
Age in years, mean (SD)	45.2 (15.4)	46.3 (15.6)	44.1 (15.1)
Race/ethnicity, *n* (%)			
White, non-Hispanic	1785 (63.7)	1041(66.4)	774 (60.9)
Black, non-Hispanic	281 (10.9)	181 (8.1)	147 (13.6)
Hispanic	333 (16.8)	186 (14.7)	147 (18.8)
Other^(a)^	226 (8.6)	66 (10.7)	160 (6.6)
Educational attainment, *n* (%)			
High school or less	381 (34.2)	208 (35.8)	273 (32.6)
Some college/associate degree	1010 (28.6)	492 (30.8)	518 (30.8)
Bachelor’s degree	718 (21.4)	299 (22.1)	419 (20.7)
Graduate degree	516 (15.8)	213 (15.7)	303 (15.9)
Household income, *n* (%)			
< $50,000	957 (40.8)	446 (36.6)	511(44.8)
$50,000–$99,000	942 (34.8)	551 (37.7)	391(32.0)
$100,000–$149,000	444 (15.2)	263 (15.3)	181 (15.1)
$150,000 +	282 (9.2)	188 (10.4)	94 (8.1)
Occupational status, *n* (%)			
Working	1921 (67.3)	1103 (70.2)	818 (64.6)
Unemployed	307 (13.8)	106 (9.0)	201(18.4)
Retired	284 (13.5)	171(12.7)	113 (12.7)
Disabled	113 (5.4)	68 (4.4)	45 (4.3)
Body mass index (kg/m^2^), mean (SD)	29.4 (7.6)	29.3 (6.8)	29.6 (8.3)
County-level poverty, *n* (%)			
Quartile 1	763 (29.7)	428 (26.6)	335 (29.6)
Quartile 2	778 (29.9)	458 (28.5)	320 (32.3)
Quartile 3	786 (29.1)	403 (32.5)	383 (24.8)
Quartile 4	295 (7.1)	157 (12.3)	238 (13.3)
Region, *n* (%)			
Northeast	382 (16.9)	216 (18.6)	166 (15.1)
Midwest	731 (20.4)	393 (19.2)	166 (21.6)
South	858 (38.2)	468 (37.5)	390 (38.9)
West	654 (20.4)	371 (24.7)	283 (24.4)

Categorical variables are presented as frequency (weighted %) and continuous variables as weighted means (SDs). ACT24 recall duration: sum of the total duration of individual physical activity behaviors reported on the previous day with at least one valid recall (4138 recalls). ^a^Other race/ethnicity includes non-Hispanics reporting other or two or more races/ethnicities

**Fig. 1:**
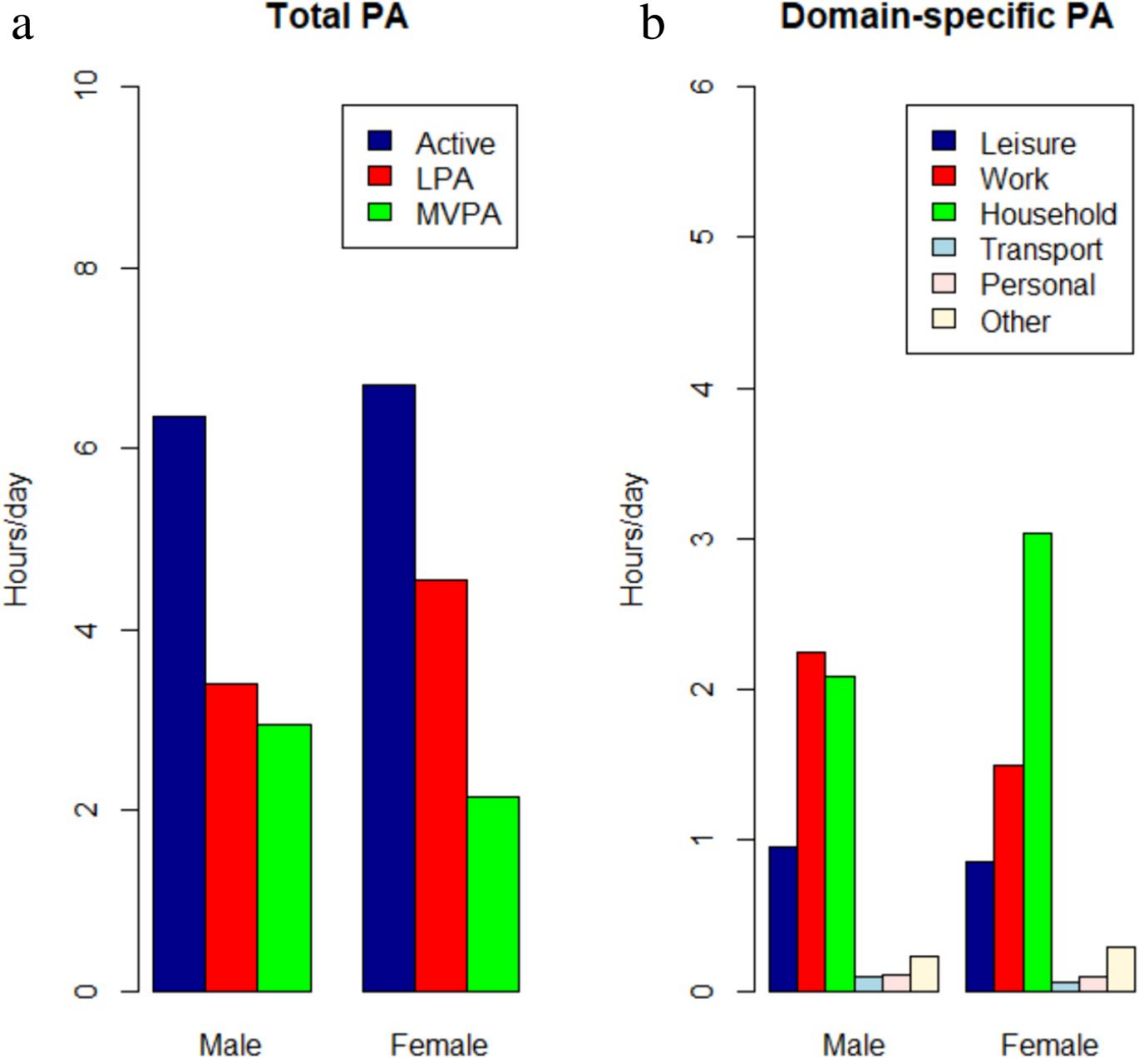
**a** Total PA (hours/day), including total active time, LPA, MVPA, stratified by gender. **b** Domain-specific PA (hours/day), including leisure, work, household, transport, personal other activities, stratified by gender

Figure 1(a) shows that participants reported a total active time of 6.5 h/d, consisting of 4.0 h/d of LPA and 6.5 h/d of MVPA, and was stratified by gender (Table S1). Figure 1(b) indicates that females spent more time on household activities than males (3.0 h/d vs 2.1 h/d), while males engaged more time in work-related activities (2.2 h/d vs 1.5 h/d) in domain-specific PA (Table S1). Additionally, the total and domain-specific PA was stratified by race/ethnicity (Figs. S1 and S2).

### Associations with Total and Intensity-Specific PA Outcomes by Race/Ethnicity and Gender

Overall, county-level segregation was not related to the PA outcomes for both NH Black and Hispanic adults (Table 2). For example, county-level segregation was not significantly associated with MVPA (*β* = 1.62, 95% CI [− 1.13, 4.37], *β* = 0.78, 95% CI [− 1.30, 2.84]) among both NH Black and Hispanic groups, respectively. Similarly, county-level segregation was not linked to any PA outcomes among NH Black and Hispanic adults (NH White adults as the reference group, Table S2).

**Table 2:** Race/ethnicity-stratified weighted multivariable linear regression models of the association between county-level segregation and PA (hours/day) in US adults

Racial residential segregation	vs All other groups	
	*β* (95% CI)	*p*-value
NH Black adults only (*n* recall = 416)		
Total active time		
Isolation Index	2.01 (− 0.77, 4.78)	0.157
LPA		
Isolation Index	0.38 (− 1.88, 2.65)	0.738
MVPA		
Isolation Index	1.62 (− 1.13, 4.37)	0.246
Hispanic adults only (*n* recall = 506)		
Total active time		
Isolation Index	0.00 (− 2.38, 2.38)	0. 999
LPA		
Isolation Index	− 0.78 (− 2.54, 0.97)	0. 380
MVPA		
Isolation Index	0.78 (− 1.30, 2.84)	0. 461

Models were adjusted for age, sex, educational attainment, occupation, income, body mass index, county-level poverty, and regions. *NH*, non-Hispanic

Among NH Black males, county-level segregation was not linked to PA outcomes, while among NH Black females, greater county-level segregation was related to engaging in more total active time (*β* = 3.54, 95% CI [0.23, 6.85], Table 3). This greater amount of total active time appeared to be derived from spending more time in LPA (*β* = 0.74, 95% CI [− 2.18, 3.66]) and MVPA (*β* = 2.08, 95% CI [− 0.25, 5.85]). Among Hispanic males, greater county-level segregation was marginally associated with less LPA (*β* =  − 2.56, 95% CI [− 5.47, 0.36]), which was offset by greater amounts of MVPA (*β* = 1.46, 95% CI [− 2.05, 4.96]), although both associations were non-significant. There were no associations between county-level segregation and PA outcomes among Hispanic females. Similar associations were found between county-level segregation and PA outcomes by race/ethnicity and gender (NH White adults as the reference group, Table S3).

**Table 3:** Race/ethnicity and gender-specific stratified weighted multivariable linear regression models of the association between county-level segregation and PA (hours/day) in US adults

Racial residential segregation	vs all other groups	
	*β* (95% CI)	*p*-value
NH Black male adults (*n* recall = 148)		
Total active time		
Isolation index	1.47 (− 5.55, 2.60)	0.474
LPA		
Isolation index	0.47 (− 2.40, 3.33)	0.746
MVPA		
Isolation index	1.94 (− 5.86, 1.98)	0.246
NH Black female adults (*n* recall = 268)		
Total active time		
Isolation index	3.54 (0.23, 6.85)	**0.036**
LPA		
Isolation Index	0.74 (− 2.18, 3.66)	0.617
MVPA		
Isolation index	2.08 (− 0.25, 5.85)	0.074
Hispanic male adults (*n* recall = 230)		
Total active time		
Isolation index	− 1.10 (− 4.73, 2.54)	0.550
LPA		
Isolation index	− 2.56 (− 5.47, 0.36)	0.085
MVPA		
Isolation index	1.46 (− 2.05, 4.96)	0.413
Hispanic female adults (*n* recall = 276)		
Total active time		
Isolation index	− 0.28 (− 3.43, 2.87)	0.860
LPA		
Isolation index	− 0.14 (− 2.40, 2.12)	0.905
MVPA		
Isolation index	− 0.14 (− 2.60, 2.31)	0.908

Models were adjusted for age, educational attainment, occupation, income, body mass index, county-level poverty, and regions

### Associations with Domain-specific PA

County-level segregation was not associated with each domain-specific PA (i.e., leisure, work, household, transport, personal, and other activities, Table 4). For example, county-level segregation was not associated with leisure PA and work PA (*β* =  − 0.70, 95% CI [− 1.68, 0.28], *β* = 1.08, 95% CI [− 0.89, 3.06], respectively) among NH Black individuals. For Hispanic adults, county-level segregation was not linked to leisure PA and work PA (*β* = 0.08, 95% CI [− 0.21, 0.38], *β* =  − 1.09, 95% CI [− 0.86, 3.03], respectively). Similarly, county-level segregation was not associated with each domain-specific PA outcome (Table S4).

**Table 4:** Race/ethnicity-stratified a stratified weighted multivariable linear regression models of the association between county-level segregation and domain-specific PA (hours/day) in US adults

Domain-specific PA	NH Black vs all other groups		Hispanic vs all other groups	
	*β* (95% CI)	*p*-value	*β* (95% CI)	*p*-value
Leisure				
Isolation index	− 0.70 (− 1.68, 0.28)	0.159	0.08 (− 0.21, 0.38)	0.576
Work				
Isolation index	1.08 (− 0.89, 3.06)	0.281	1.09 (− 0.86, 3.03)	0.272
Household				
Isolation index	0.72 (− 0.44, 1.88)	0.221	− 0.52 (− 1.50, 0.45)	0.292
Transport				
Isolation index	0.05 (− 0.25, 0.36)	0.709	− 0.05 (− 0.23, 0.13)	0.580
Personal				
Isolation index	0.21 (− 0.06, 0.47)	0.124	− 0.03 (− 0.54, 0.60)	0.920
Other				
Isolation index	0.42 (− 0.39, 1.23)	0.311	0.15 (− 0.48, 0.79)	0.638

Models were adjusted for age, sex, educational attainment, occupation, income, body mass index, county-level poverty, and regions

## Discussion

In this study, we aimed to address gaps in the existing literature by examining the relationship between county-level racial/ethnic residential segregation and various PA outcomes in a representative sample of US adults. We found that NH Black females living in the more segregated counties had greater total active time, which may be driven by greater work- and household-related activities. Lastly, we found no significant associations between more segregated counties and each domain-specific PA outcome, including leisure, work, household, transport, personal, and other activities. Further work on gender-specific associations between county-level segregation and PA could help increase our understanding of structural influences on behavior and design and develop interventions to increase PA.

Our finding of no association between county-level residential segregation (NH Blacks vs other groups) and PA outcomes, including LPA, MVPA, and total active time, was consistent with previous studies [16, 29]. One study using the 2001 Behavioral Risk Factor Surveillance System (BRFSS) found that highly segregated Black-White neighborhoods (based on the dissimilarity index) were not associated with leisure time PA (i.e., based on 30 min of MPA per day at least 5 days/week or 20 min of VPA per day at least 3 days/week) among Black adults only [16]. However, one study using the 1999–2004 NHANES indicated that highly segregated predominantly Black neighborhoods (based on neighborhood racial composition at the census tract level) were associated with lower odds of being physically active compared to Whites in White neighborhoods [15]. Overall, our study and other studies [16, 29] found no significant association between county-level segregation and PA. The inconsistent findings from one study to the next may be due to variations in the measurement of segregation, as neighborhood racial composition [15] served as an indicator of residential segregation compared to our study of isolation segregation indices. Future research should investigate these inconsistencies deeper to better understand the relationship between residential segregation and PA.

Similarly, our finding indicates that residential segregation for Hispanic adults (vs all other adults) was not related to any PA outcomes, which was inconsistent with the previous study [30]. A study using the 2000 BRFSS data found that Hispanic adults residing in highly segregated metropolitan statistical areas (based on the dissimilarity index) had 18% lower leisure-time PA levels (any exercise in the past month [yes/no]) than those residing in less segregated areas [30]. Several factors might account for differences between our study and the previous study [30]. First, our sample size of Hispanic participants (*n* = 333) is much smaller than the previous study (8785 participants) [30], which might lead to limited statistical power and an ability to identify weaker or null associations. In addition, our PA outcome variables included LPA, MVPA, and total active time, incorporating daily activities undertaken at home, work, and during transportation, as opposed to the single PA measure utilized in the previous study [30]. Future studies need to replicate this line of research in other datasets.

The gender-specific analysis indicated significant and marginally significant associations of higher segregation with greater total active time and MVPA among NH Black female adults, respectively. This finding was not consistent with the findings from the previous study [31]. The previous study found that NH Black females were more physically active in neighborhoods perceived as predominantly white communities and less likely to engage in leisure-time PA in neighborhoods perceived as predominately black communities [31]. The authors explained that NH Black women were more likely to participate in leisure-time PA in safe and well-resourced neighborhoods, typically found in White neighborhoods [31]. The inconsistency in findings may arise from methodological and sampling differences. For example, the sample of the previous study [31] consisted of highly educated, mostly full-time participants, while our sample is more diverse (high school or less: 34%; employed: 67%). One explanation for the positive association from our study may be the formation of tight-knit communities, particularly among Black female adults. Such communities could provide social support networks that may buffer the adverse effects of residential segregation, such as exposure to discrimination and residing in less safe neighborhoods [18]. This sense of perceived safety, acceptance, and belonging among NH Black women may foster social cohesion, thereby encouraging them to engage in more PA. Further research is needed to confirm this association between segregation and PA outcomes among NH Black females residing in highly segregated counties.

Lastly, our findings indicated that county-level residential segregation was not associated with domain-specific PA, including leisure, work, household, transport, personal, and other activities, among the NH Black and Hispanic adults. To our acknowledge, this is the first study to examine the relationship between segregation and domain-specific PA. Despite the lack of a significant association between county-level segregation and each domain-specific PA, distinct variations were observed in PA behaviors across different domains and racial/ethnic groups [32]. For example, although county-level segregation was not associated with leisure and work-related PA behaviors for NH Black individuals, it showed a negative pattern with leisure PA and a positive pattern with work-related PA. An opposite association for leisure PA was observed among Hispanic adults, but consistent for work PA. This highlights the other factors, such as the built environment characteristics and social norms within various communities. Future studies should aim to elucidate these complexities, contributing to a more comprehensive understanding of how residential segregation is related to domain-specific PA.

The strengths of this study included its utilization of racially and ethnically diverse study population, enhancing the potential for nationally representative and more generalizable findings. Secondly, ACT24 has demonstrated reasonable accuracy and validity in estimating PA, enhancing the reliability of our measurements. Furthermore, this study addressed a crucial gap by investigating the role of gender in the associations between racial/ethnic residential segregation and various PA and domain-specific PA outcomes. This critical component has been previously understudied. Furthermore, our residential segregation (isolation index) was constructed using county-level measurements rather than smaller geographic units, such as census tracts. While using smaller units could capture more nuanced neighborhood characteristics related to individuals’ PA behavior, measuring segregation at the county level offers a broader geographic scope and provides more reliable data compared to smaller units [33].

There are several limitations in our study. First, the analysis was based on self-reported measures of PA, which may introduce recall and social desirability biases [24, 34]. However, a previous study demonstrated that ACT24 had been validated with objective PA measures, demonstrating higher correlations and providing better performance than traditional questionnaires in large epidemiologic studies. Additionally, the study design was cross-sectional, limiting our ability to infer causal relationships. Future research could employ longitudinal designs and objective measures of PA to further explore the relationship between racial/ethnic residential segregation and PA. A fourth limitation is lack of detailed information about employment characteristics, specific job types involve different levels of PA [35], and job types could differ for people living in more or less segregated neighborhoods. Lastly, our data is nationally representative and had a relatively small sample size for minority groups, specifically NH Black and Hispanic adults. Therefore, the generalizability of our findings may be limited for these specific racial and ethnic groups. To enhance the external validity for future studies, it is crucial to include more minority participants, and oversample other minority groups that encompass a broader representation of racial and ethnic backgrounds, including Asians and other populations, and to perform detailed qualitative and quantitative analysis on those understudied minoritized individuals to elucidate how structural factor, such as segregation may be contributing to their PA levels. This would enable a more comprehensive understanding of how residential segregation may be related to PA outcomes across different racial and ethnic groups. Future studies should employ more detailed qualitative and quantitative analyses to delve deeper into the nuances of specific minority communities and contribute to advancing our understanding of the interplay between segregation and PA outcomes among diverse racial and ethnic groups.

## Conclusions

In conclusion, our study contributes to expanding the current literature on the influence of residential segregation and PA among US adults. The results indicated that NH Black females residing in segregated neighborhoods had more total active time. Additionally, it is important to acknowledge that these relationships may vary among NH Black and Hispanic populations. Future research should examine a more complete set of environmental, contextual, and individual influences on PA and domain-specific PA.

## Disclaimer

The views of this study are those of the authors and do not necessarily represent the views of the NIMHD, NCI, the NIH, or the US Department of Health and Human Services.

## Supplementary Information

Below is the link to the electronic supplementary material.

Supplementary file1 (DOCX 131 KB)

## Data Availability

Data described in the manuscript, code book, and analytic code will be made available upon request pending application and approval.
