# Supplementary material for: County-level Racial/Ethnic Residential Segregation and Physical Activity Behavior among US Adults
Source: J Urban Health. 2024 Sep 10;102(1):61–71. doi: 10.1007/s11524-024-00913-4 (PMC11865418; doi:10.1007/s11524-024-00913-4)
Supplement: Supplementary file 1 — Supplementary file1 (DOCX 131 KB) [file 11524_2024_913_MOESM1_ESM.docx]

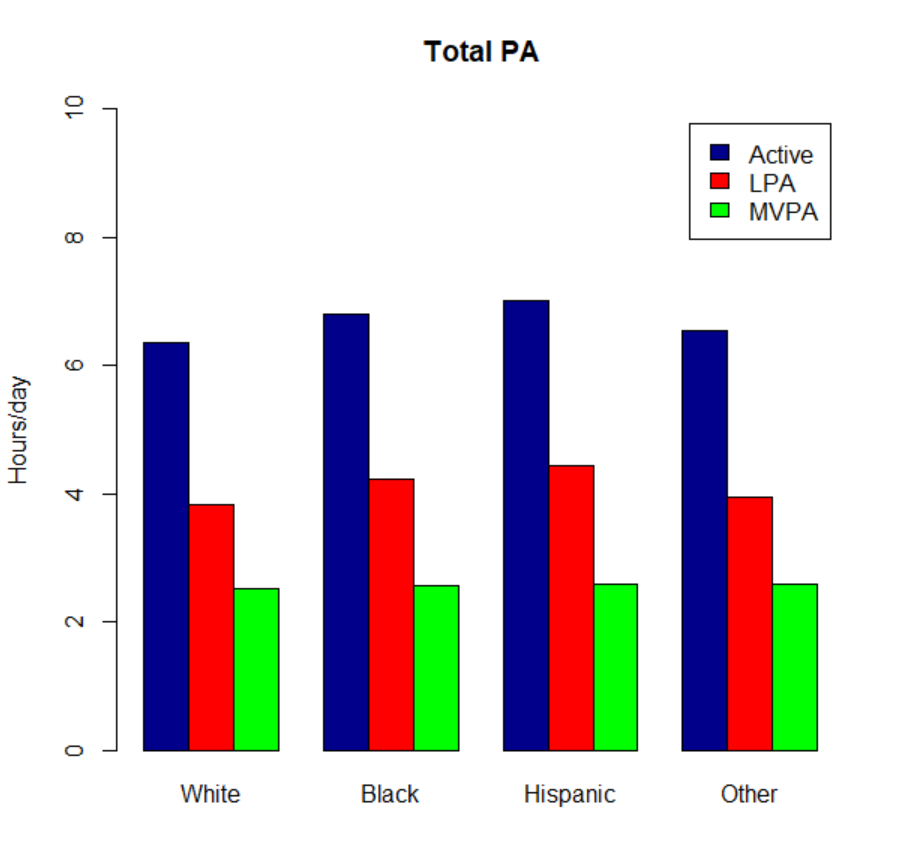


S1 fig. Total PA (hours/day), including total active time, LPA, MVPA, stratified by Race/Ethnicity. Other refers to non-Hispanics reporting other or two or more races/ethnicities.


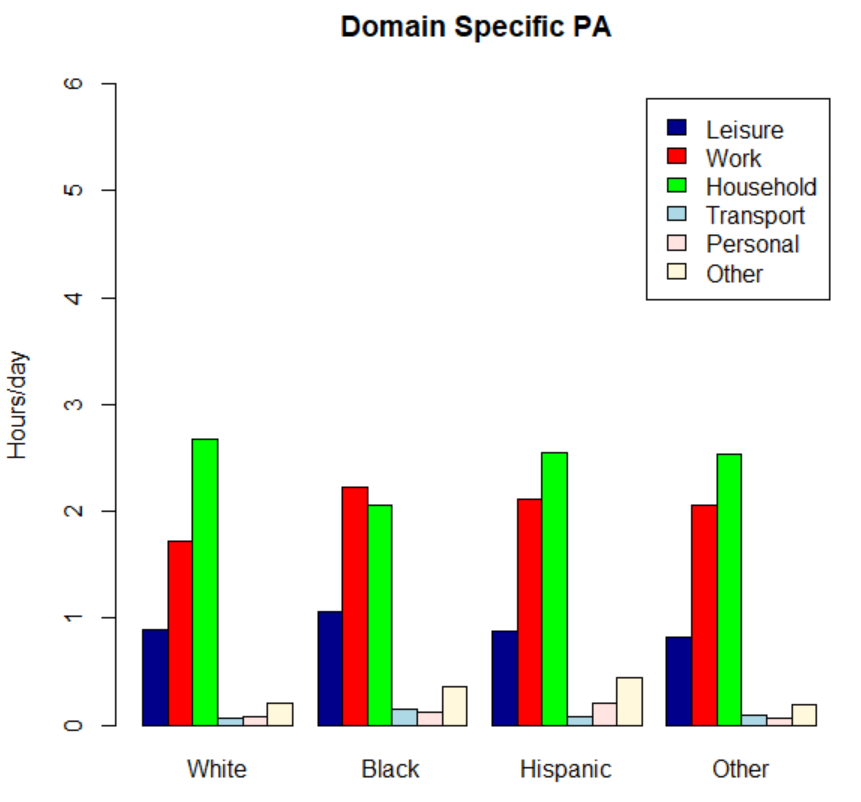


S2 fig. Domain-specific PA (hours/day), including personal, leisure, work, household, transport, other activities, stratified by race/ethnicity. Other refers non-Hispanics reporting other or two or more races/ethnicities.

| S1 Table. Weighted descriptive characteristics of physical activity (PA, hours/day) | | | |
| --- | --- | --- | --- |
| **Characteristic** | **All** | **Male** | **Female** |
| **Total PA, mean (SD)** |  |  |  |
| LPA | 3.98 (2.8) | 3.39 (2.7) | 4.55 (2.8) |
| MVPA | 2.55 (3.4) | 2.95 (3.7) | 2.15 (3.0) |
| Active time-use | 6.53 (3.1) | 6.34 (3.8) | 6.70 (3.6) |
| **Domain-Specific PA, mean (SD)** |  |  |  |
| Leisure | 0.10 (1.4) | 0.95 (1.5) | 0.85 (1.3) |
| Work | 1.87 (3.2) | 2.24 (3.5) | 1.50 (2.8) |
| Household | 2.58 (2.6) | 2.09 (2.4) | 3.04 (2.7) |
| Transport | 0.08 (0.4) | 0.04 (0.2) | 0.05 (0.2) |
| Personal | 0.09 (1.2) | 0.10 (1.3) | 0.06 (0.8) |
| Other | 0.13 (1.4) | 0.14 (1.4) | 0.13 (1.0) |

Variables as weighted means (SDs)

| S2 Table. Race/ethnicity-stratified weighted multivariable linear regression models of the association between county-level segregation and PA (hours/day) in US adults | | |
| --- | --- | --- |
| Racial Residential Segregation Indices by Race/Ethnic Groups | vs White | |
|  | β (95% CI) | p-value |
| **NH Black adults (n recall = 416)** |  |  |
| **Total active time** |  |  |
| Isolation Index | 1.74 (-0.60, 4.10) | 0.145 |
| **LPA** |  |  |
| Isolation Index | 0.24 ( -1.76, 2.23) | 0.817 |
| **MVPA** |  |  |
| Isolation Index | 1.51 (0.83, 3.85) | 0.205 |
| **Hispanic adults (n recall = 506)** |  |  |
| **Total active time** |  |  |
| Isolation Index | -0.44 (-2.57, 1.68) | 0.681 |
| **LPA** |  |  |
| Isolation Index | -0.70 (-2.28, 0.87) | 0.387 |
| **MVPA** |  |  |
| Isolation Index | 0.25 (1.68, 2.19) | 0.797 |
| *Note:* Models were adjusted for age, sex, educational attainment, occupation, income, body mass index, county-level poverty, and regions. | | |

| S3 Table. Race/Ethnicity and gender-specific weighted multivariable linear regression models of the association between county-level segregation and PA (hours/day) in US adults | | | | | |
| --- | --- | --- | --- | --- | --- |
| Racial Residential Segregation Indices by Race/Ethnic and Gender Groups | NB Black vs NH White | |  | Hispanic vs NH White | |
|  | β (95% CI) | p-value |  | β (95% CI) | p-value |
| **NH Black Male (n recall=148)** | | | **Hispanic Male (n recall=230)** | | |
| **Total active time** |  |  | **Total active time** |  |  |
| Isolation Index | -1.75 ( -5.77, 2.27) | 0.390 | Isolation Index | -2.37 (-5.76, 1.02) | 0.169 |
| **LPA** |  |  | **LPA** |  |  |
| Isolation Index | 0.61 (-2.15, 3.37) | 0.661 | Isolation Index | **-2.65 (-5.34, 0.034)** | **0.053** |
| **MVPA** |  |  | **MVPA** |  |  |
| Isolation Index | -2.36 ( -6.20, 1.49) | 0.226 | Isolation Index | 0.28 (-3.31, 3.88) | 0.876 |
| **NH Black Female (n recall =268)** | | | **Hispanic Female (n recall =276)** | | |
| **Total active time** |  |  | **Total active time** |  |  |
| Isolation Index | **3.15 (0.51, 5.78)** | **0.019** | Isolation Index | **3.54 (0.23, 6.85)** | **0.036** |
| **LPA** |  |  | **LPA** |  |  |
| Isolation Index | 0.41 (-2.05, 2.86) | 0.745 | Isolation Index | 0.74 (-2.18, 3.66) | 0.617 |
| **MVPA** |  |  | **MVPA** |  |  |
| Isolation Index | **2.74 (0.19, 5.29)** | **0.035** | Isolation Index | **2.08 ( -0.25, 5.85)** | **0.072** |
| ***Note:*** Models were adjusted for age, sex, educational attainment, occupation, income, body mass index, county-level poverty, and regions. | | | | |  |

| S4 Table. Race/ethnicity-stratified weighted multivariable linear regression models of the association between county-level segregation and Domain-specific PA (hours/day) in US adults | | | | | |
| --- | --- | --- | --- | --- | --- |
| Domain-Specific PA |  |  |  | |  |
|  | NH Black vs White |  | Hispanic vs White | |  |
|  | β (95% CI) | p-value | β (95% CI) | p-value | |
| **Leisure** |  |  |  |  | |
| Isolation Index | -0.68 (-1.54, 0.18) | 0.124 | 0.11 (-0.21, 0.38) | 0.386 | |
| **Work** |  |  |  |  | |
| Isolation Index | 1.27 (-0.39, 2.92) | 0.133 | 0.66 (-1.10, 2.42) | 0.459 | |
| **Household** |  |  |  |  | |
| Isolation Index | 0.63 (-0.37, 1.64) | 0.217 | -0.56 (-1.48, 0.36) | 0.233 | |
| **Transport** |  |  |  |  | |
| Isolation Index | -0.01 (-0.32, 0.31) | 0.956 | -0.01 (-0.12, 0.11) | 0.904 | |
| **Personal** |  |  |  |  | |
| Isolation Index | 0.17 (-0.08, 0.42) | 0.174 | 0.01 (-0.55, 0.58) | 0.961 | |
| **Other** |  |  |  |  | |
| Isolation Index | 0.42 (-0.43, 1.01) | 0.426 | 0.02 (-0.48, 0.64) | 0.646 | |
| ***Note:*** Models were adjusted for age, sex, educational attainment, occupation, income, body mass index, county-level poverty, and regions | | | | | |
